# Intestinal Transit in Early Moderate Parkinson's Disease Correlates with Probable RBD: Subclinical Esophageal Dysmotility Does Not Correlate

**DOI:** 10.1155/2022/4108401

**Published:** 2022-07-15

**Authors:** Casper Skjærbæk, Karoline Knudsen, Martin Kinnerup, Kim Vang Hansen, Per Borghammer

**Affiliations:** ^1^Department of Nuclear Medicine and PET, Aarhus University Hospital, Palle Juul-Jensens Boulevard 165, DK-8200 Aarhus N, Aarhus, Denmark; ^2^Department of Neurology, Aarhus University Hospital, Palle Juul-Jensens Boulevard 165, DK-8200 Aarhus N, Aarhus, Denmark; ^3^Department of Regional Psychiatry Randers, Psychiatry of Central Denmark Region, Dronningborg Blvd. 15, DK-8930 Randers, Randers, Denmark

## Abstract

**Background:**

Nonmotor symptoms, including constipation and dysphagia, are very common in Parkinson's disease (PD) and Lewy pathology is widespread in the gastrointestinal tract, particularly in the lower esophagus. Constipation and REM sleep behavior disorder (RBD) may present prior to clinical diagnosis. Yet, little is known about esophageal dysfunction and its connection to constipation in early PD.

**Objective:**

This study aimed to investigate esophageal and colonic transit in early moderate PD and to study correlations between symptoms and objective measures.

**Methods:**

Thirty early moderate PD patients and 28 healthy controls (HC) were included in this cross-sectional study. Esophageal transit times were determined by esophageal scintigraphy and colonic transit times by CT after radio-opaque marker ingestion. Olfaction tests, clinical evaluation, and nonmotor questionnaires were also performed.

**Results:**

Distal esophageal transit times and colonic transit times were both significantly prolonged in the PD group compared to HC (*p* < 0.05 and*p* < 0.01, respectively) and a moderate-strong positive correlation was found between colonic transit time (CTT) and RBDSQ score (*r* = 0.61,*p* < 0.001). Significant correlations were also found between CTT and SCOPA-AUT scores as well as between CTT and ROME III functional constipation scores.

**Conclusion:**

Colonic transit correlates with probable RBD and is more severely prolonged in early moderate PD than is the distal esophageal transit time.

## 1. Introduction

Nonmotor symptoms (NMS) are very common in Parkinson's disease (PD) including constipation, dysphagia, and sleep disturbances [1]. Constipation and REM sleep behavior disorder (RBD) can develop several years prior to the PD diagnosis [2, 3]. The motor symptoms of PD manifest as Lewy pathology in the substantia nigra accumulates, and RBD is expected to be a result of pontine pathology [4]. Lewy bodies and neuronal degeneration in the gastrointestinal tract presumably contribute to gastrointestinal NMS and are present several years before PD diagnosis in some patients [5]. In manifest PD, the pathology is widespread throughout the gastrointestinal tract and most pronounced in the vagus-innervated distal esophagus and stomach [6–8]. Interestingly, this rostrocaudal gradient of pathology does not align with the gut-related burden of gastrointestinal symptoms. In prodromal and early PD, constipation and prolonged colonic transit time are frequent, whereas gastric emptying time is normal in most patients [9]. Also, dysphagia is rarely seen in prodromal PD but quite common in later disease stages [10] underlining the importance of investigating these NMS separately.

Despite widespread pathology of the distal esophagus in PD, biomarkers of esophageal function have received little attention [11]. Thus, it is largely unknown whether subclinical esophageal dysfunction is present in early PD. Furthermore, the relationship between esophageal and colonic dysfunction in PD has not previously been investigated using objective measures.

This study aimed to investigate esophageal dysfunction in early moderate PD using scintigraphy and to examine potential correlations among subjective symptoms and objective markers of autonomic dysfunction including esophageal and colonic dysfunction. We hypothesized that colonic and distal esophageal transit times are prolonged in PD compared to healthy controls (HC) and that prolonged transit times are more frequent findings than corresponding subjective symptoms of dysphagia and constipation.

## 2. Methods

### 2.1. Study Population and Ethics Statement

In this case-control study, we included 30 early to moderate stage PD patients and 28 HC matched on age and sex. The study was approved by the Central Denmark Region Committee on Health Research Ethics (1-10-72-195-18). All participants provided informed written consent according to the Declaration of Helsinki.

Inclusion criteria were 50–85 years of age and patients with a PD diagnosis set by a movement disorder specialist according to MDS Clinical Diagnostic Criteria for Parkinson's Disease [12]. Patients and healthy controls were recruited using advertisements in local newspapers and bulletins from the local Parkinson's disease patient associations (26 PD, 27 HC) or directly from the outpatient clinic at the Department of Neurology at Aarhus University Hospital (4 PD, 1 HC). Patients recruited from newspapers and bulletins were from different outpatient clinics and private neurology services across Jutland. Most HC were spouses or friends with a PD patient. Exclusion criteria were patients with neurological disease, major gastrointestinal disorders, prior gastrointestinal surgery (except appendectomy, minor hernia repairs, and diagnostic biopsies), structural abnormalities of the gastrointestinal tract, and major systemic disorders including diabetes and renal, heart, or liver failure. Neither patients nor HC was selected based on the presence or absence of dysphagia or gastrointestinal symptoms.

The PD diagnosis was supported by pathological ^123^I-FP-CIT-SPECT scans in 13 patients. All PD patients received anti-Parkinsonian medication. All medications including laxatives were continued throughout the study. All data were analyzed blinded to the clinical category.

### 2.2. Esophageal Scintigraphy

Esophageal motility was investigated using scintigraphy performed on Siemens Symbia T16 (Siemens AG, Erlangen, Germany) with a sample rate of 8 frames per second for both the anterior and posterior gamma cameras. Subjects were in a fasted state for solids and liquids for >8 hours, but prior to scintigraphy, all participants ingested a glass of water. The examination was performed in the supine position to eliminate gravitational effects, and the cameras were positioned to visualise the mouth, esophagus, and proximal stomach. A bolus of 3–5 mL mixture of water, thickening agent (ThickenUp, Nestlé Health Science), and 99^m^Tecnetium (NanoCis, Cis Bio International) was dispensed into the subject's mouth using a 5 mL syringe. Participants were instructed to retain the bolus in the mouth until a signal was given and thereafter swallow the bolus in one gulp. Each subject had four radioactive swallows receiving a total of 19.2–24.6 MBq 99^m^Tc. Four different mixtures were used to test if transit time was affected by bolus viscosity and volume. All participants received two 3 mL boluses (mixture of 100 mL water, 6 g thickening agent, intermediate viscosity similar to jam). Additionally, half of the participants received two lower viscosity 3 mL boluses (100 mL water, 3 *g* thickening agents), while the other half received two high viscosity 5 mL boluses (50 mL water, 6 g thickening agent). Subjects were allowed to dry swallow after swallowing the bolus. Before swallowing the first bolus, one or two test swallows with 5 mL of water were performed. Four minutes separated each swallowed bolus, and before the next bolus, the subject was given 5 mL of water two times to minimize remaining activity in the mouth and esophagus.

Subjects were excluded post hoc from the esophageal scintigraphy dataset, if computed tomography (CT) gave rise to suspicion of hiatal hernia, defined as a dilation of the esophageal hiatus of >2.5 cm in combination with an abnormally dilated, air-filled lumen proximal to the diaphragm. The location of the gastroesophageal junction was determined prior to the analysis of transit times based on CT and the summed esophageal scintigraphy image.

### 2.3. Dynamic Analysis of Esophageal Scintigraphy

Using dedicated software (PMOD Technologies), each frame from the posterior camera was flipped 180 degrees and added to the anterior camera data and smoothed using a 4 mm Gaussian filter.

**Figure 1:**
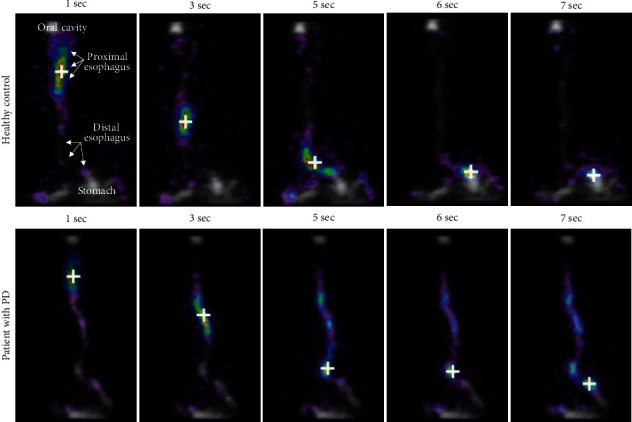
Serial scintigraphy images illustrating esophageal transit of a healthy control (normal transit) and a PD patient (prolonged transit). In grey is the summed activity of the whole recording, and in colors (purple to red) is shown the activity second by second. The white crosshair marks the algorithm-based estimate of the center of the bolus.

An automated software algorithm was developed to track bolus location in every frame of each dynamic recording (Figure 1). Briefly, bolus coordinates were identified as the midpoint of the lowermost area of the esophageal signal with an area equal to or greater than 12 square millimeters in the processed frame. Recordings were considered eligible for analysis if the algorithm identified an unequivocal bolus traveling throughout the distal esophagus.

Region of interest (ROI) analyses were performed by placing 10 ROIs (each 20 × 30 mm) evenly from the upper esophagus to the gastroesophageal junction. The time of peak activity in each ROI was determined and transit time was defined as the difference in time of peak activity. Additionally, a large upper-ROI covering the upper 3 ROIs and a large distal-ROI covering the distal 3 ROIs were drawn, and the length of time from 20% of peak activity was reached until enough activity has passed to again reach 20% of peak activity was noted (Supplementary Materials).

### 2.4. Colonic Transit Time and Colonic Volume

After scintigraphy, colonic transit time (CTT) was evaluated using radio-opaque markers (ROM) [13]. Briefly, one capsule containing 10 ROMs was ingested each morning for six consecutive days (60 ROMs total). Low-dose CT was performed on a Siemens Symbia T16 (Siemens AG) 24–28 hours after ingestion of the last capsule. The number of ROM was counted on CT, and the total CTT was calculated using the equation: CTT = (ROM (total number) + 5)/10 days [13].

### 2.5. Clinical Assessment and Questionnaires

Patients' motor function and disease stage was evaluated using the Movement Disorder Society-Unified Parkinson's Disease Rating Scale (MDS-UPDRS) Part III and the Hoehn and Yahr scale (H&Y) [14]. The levodopa equivalent daily dose (LEDD) was calculated [15]. The supine resting blood pressure was measured after 15 minutes of rest and orthostatic hypotension was defined as a systolic blood pressure drop of 20 mmHg or diastolic blood pressure drop of 10 mmHg within the first three minutes of standing. The cognitive function was screened using the Montreal cognitive assessment (MoCA) [16]. Olfactory function was assessed using Sniffin' Sticks 16-item identification test [17].

Overall burden of NMS was evaluated by SCOPA-AUT [18]. Lower gastrointestinal symptoms were assessed by the ROME III functional constipation questionnaire [19]. Symptoms of dysphagia and esophageal disorders were assessed using the Munich dysphagia test-Parkinson's disease (MDT-PD) [20] and the ROME III functional esophageal disorders module [19]. The MDT-PD was evaluated as an unweighted total and as a weighted score returned by the web application that by using weights for each variable based on binary logistic regression weights each item based on aspiration risk [20]. The RBD screening questionnaire (RBDSQ) was used with a cutoff of ≥5 to identify probable RBD (pRBD) [21].

### 2.6. Statistical Analyses

Statistical analyses were performed using STATA 13 (Stata Corp.) and Prism 8 (GraphPad Software). To test for differences in demographic and clinical data, esophageal transit times and questionnaires, unpaired two-way *t*-tests, nonparametric tests, or Fisher's exact test were used as appropriate. Transit time outliers were identified using the ROUT method (*Q* value 0.1%). Correlations were tested using Spearman and Pearson rank tests. Receiver-operating characteristic (ROC) analyses were performed to establish optimal cutoff thresholds for separating the PD patients from controls with regard to colonic and esophageal transit times.

## 3. Results

Demographic and clinical data are given in Table 1. No differences were seen in sex distribution, age, height, and body mass index (BMI). The PD group had significantly decreased olfaction and MoCA scores and higher rates of orthostatic hypotension compared to controls. Eighteen PD patients and one HC had pRBD. Of note, this one HC with pRBD scored 5 on RBDSQ and 11 on Sniffin' Sticks (1 SD below the mean for HC) and had 27 ROMs on CT (above normal range for men). Questionnaire results regarding autonomic dysfunction are given in Table 1. The overall burden of autonomic dysfunction measured by the SCOPA-AUT was significantly higher in PD patients (<0.0001) and symptoms of constipation according to ROME III criteria were more prevalent in PD patients (<0.0001).

### 3.1. Esophageal and Colonic Transit Times

Two PD and six HC had hiatal hernia on CT and were excluded from the esophagus scintigraphy dataset. In some recordings, the bolus was not visualise clearly through the entire esophagus, and these recordings were excluded from analysis. In one HC, the quality of all recordings was insufficient for analysis. Thus, the final scintigraphy dataset comprised 26 PD and 21 HC.

**Figure 2:**
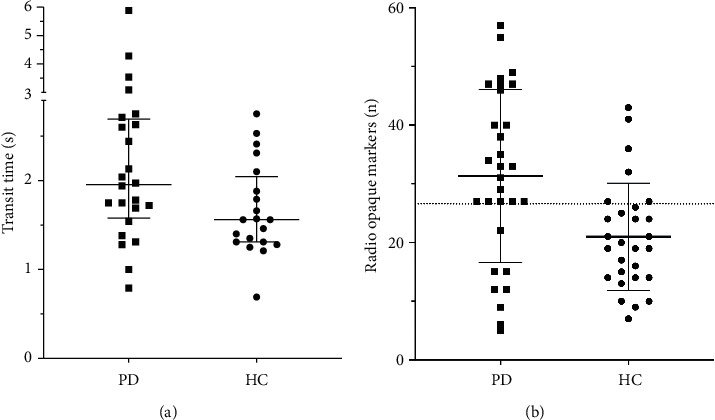
(a) Transit time through the distal four centimeters of the esophagus (*p* < 0.017). Bars indicate median with interquartile range. (b) Total number of retained radio-opaque markers (ROM) in the gastrointestinal tract (*p*=0.0021). Bars indicate mean ± SD. Dotted line indicates a tentative cutoff for prolonged intestinal transit.

No detectable differences in transit times were found between tested viscosities. Thus, the mean of all eligible recordings from each subject was used in the analyses of esophageal transit. Esophageal transit times are given in Table 1. The transit through the distal four centimeters of the esophagus as measured by the automated software algorithm was significantly prolonged in the PD group (Figure 2(a), *p*=0.011), and without exclusion of outliers, the between-group difference remained significant (*p*=0.016). Using the ROI-based method, the transit through the distal esophagus did not reach statistical significance, but the distal transit time trended towards prolongation in the PD group (*p*=0.075). No differences in upper esophageal transit times were found between groups and the total esophageal transit time was similar between groups using both methods.

Constipation defined as <3 bowel movements per week was present in 12 PD and 1 HC, while 21 PD and 5 HC had objectively prolonged gastrointestinal transit using cutoffs of >24 ROMs for males and >28 for females [22]. The total number of retained ROMs and corresponding CTTs were significantly higher in the PD group (Figure 2(b), *p*=0.0021). ROC analysis based on total number of retained ROMs yielded an optimal ROMs cutoff count of >26.5 markers translating into a sensitivity of 73% and a specificity of 79%, whilst ROC analysis of the esophageal transit time through the distal four centimeters yielded an overall sensitivity of 79% and specificity of 62% using a cutoff of >1.7 seconds. When prioritizing specificity, the optimal cutoff was 2.6 seconds yielding a specificity of 91% and a sensitivity of 43%.

### 3.2. Correlations

Distal esophageal transit time did not correlate with the total number of ROMs nor with symptoms of dysphagia as measured by the sum and aspiration-weighted MDT-PD score and by the ROME III functional esophageal disorders (*p* > 0.05). A negative correlation between distal esophageal transit and BMI (*p*=0.046) was found. All measures of distal esophageal transit correlated significantly.

**Figure 3:**
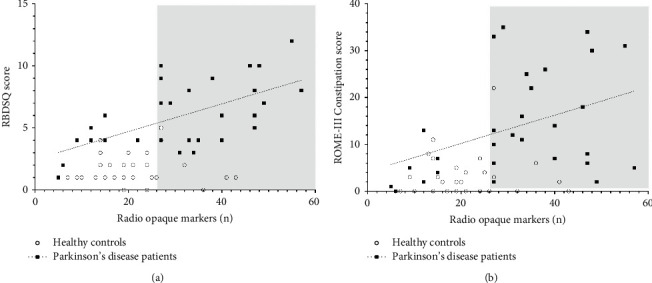
(a) Association between RBDSQ score and total number of retained radio-opaque markers (ROM) in the gastrointestinal tract. The dotted line indicates the linear regression line (linear regression slope for PD patients of 0.1119, *r* = 0.61, *p*=0.0003). The grey box indicates a tentative cutoff for prolonged intestinal transit time. (b) Association between ROME III functional constipation score and total number of retained radio-opaque markers (ROMs) in the gastrointestinal system. The dotted line indicates the linear regression line (linear regression slope for PD patients of 0.30, *r* = 0.40, *p*=0.029) in the PD patient group. The grey box indicates a tentative cutoff for prolonged intestinal transit time.

Significant positive correlations were seen between total number of ROMs and RBDSQ score (Figure 3(a), *p*=0.0003) and between total ROM and the ROME III functional constipation score (Figure 3(b), *p*=0.038). Also, a significant correlation was found between the total number of ROMs and the total SCOPA-AUT score (*p*=0.032).

Levodopa equivalent daily dose (LEDD) correlated positively with symptom duration (*p*=0.002) and disease duration (*p*=0.002) but did not correlate with colonic transit time, use of laxatives, or MDS-UPDRS Part III. Laxatives were taken by 19 patients and 7 HC.

## 4. Discussion

Here, we compared objective and subjective measures of esophageal and colonic dysfunction in PD patients and HCs. Distal esophageal transit times were significantly prolonged in PD patients and correlated with decreased BMI. Colonic transit was markedly delayed in the PD group, and interestingly, a robust positive correlation was detected between colonic transit and symptoms of RBD.

### 4.1. Esophageal Dysfunction and Symptoms of Dysphagia

We studied a group of unselected PD patients using esophageal scintigraphy and applied a newly developed and automated software algorithm capable of detecting the transit of the swallowed bolus on a per frame basis to demonstrate increased transit time through the distal 4 centimeters of the esophagus. Previously, esophageal scintigraphy was performed in a highly selected cohort of 18 PD patients of which 13 had clear symptoms of dysphagia [23]. A very pronounced difference in total esophageal transit time (14.46 s vs. 7.45 s) and a moderate difference in distal esophageal transit (8.81 vs. 6.14) were found compared to healthy controls [23]. However, that study did not exclude patients with hiatal hernia, the analysis methodology were incompletely explained, and individual data points are not presented in the study. Thus, it is difficult to compare those results with our findings.

Interestingly, esophageal manometry has identified alterations in basal pressure and relaxation of the distal esophageal sphincter in about 25% of patients with no significant difference between early and later disease stages. This aligns well with our findings, as using a cutoff for distal esophageal transit of 2.6 seconds identifies 12 patients and 3 controls as having prolonged transit corresponding to a specificity of 91% and a sensitivity of 43%. Both methods point to distal esophageal dysfunction in a substantial minority of early stage patients, which contrasts with the general notion that dysphagia in PD is mainly a feature of advanced disease [10].

Weight loss and increased risk of aspiration pneumonia, both leading causes of death in PD [24, 25], are possible consequences of dysphagia. Treatment of dysphagia in PD is predominantly aimed at oropharyngeal difficulties [26], but the underlying mechanisms of esophageal and oropharyngeal dysfunction are inherently different [24, 27]. The somatic motor nuclei and nerves innervating the upper esophagus display little Lewy pathology [28]. Conversely, the distal esophagus smooth muscle is innervated by the dorsal motor nucleus of vagus, which contains marked Lewy pathology in most PD cases [28, 29], and Lewy pathology is also abundant in the peripheral nervous plexus of the distal esophagus [28].

In the present study, no significant differences were demonstrated between PD and HC in the ROME III functional esophageal disorders or the weighted MDT-PD, whereas the unweighted MDT-PD scores were significantly different between PD cases and HC. Weighting of the MDT-PD scores is aimed at determining the risk of aspiration for which fiberoptic endoscopic evaluation of swallowing (FEES) is considered the golden standard [20, 24]. However, this technique is not capable of exploring esophageal dysfunction which may explain the lack of correlation between esophageal dysfunction and questionnaire results. This suggests that distal esophageal dysmotility is not associated with increased risk of aspiration. However, distal esophageal dysfunction in PD might still be clinically relevant as the distal esophageal transit time correlated negatively with BMI in the early moderate PD patients suggesting that distal esophageal dysmotility might contribute to early weight loss.

### 4.2. Colonic Dysfunction and RBD

Questionnaires are often used to measure NMS, but has been demonstrated to underestimate the prevalence of objectively measured dysfunction in a meta-analysis of oropharyngeal dysphagia [30] as well as of constipation [22]. A prominent issue in evaluating subjective symptoms is the availability of multiple different questionnaires and definitions of each NMS [22].

A moderate-strong correlation was found between colonic transit time and RBDSQ scores, and a less robust but still significant correlation was seen between the ROME III functional constipation scores and colonic transit time.

Objective colonic dysfunction has previously been linked to RBD, and extensive gastrointestinal Lewy pathology has been demonstrated in patients with isolated RBD (iRBD) [31], and nearly all subjects with iRBD convert to synucleinopathy within a 15-year period [3]. Accordingly, a recent study demonstrated that de novo PD patients with premotor RBD showed substantially longer colonic transit times than PD patients without RBD [32], and it has been proposed that iRBD is a marker of a body-first subtype of PD [32]. Interestingly, the present study suggests that the RBDSQ is a better predictor of objective colonic dysfunction than is the subjective symptoms of constipation. This finding supports the link between colonic dysfunction and symptoms of RBD and underlines that self-reported symptoms are incomplete measures of objective dysfunction. However, as the present study only included patients with manifest PD, further studies are needed to clarify whether these findings also apply to patients with iRBD.

### 4.3. Limitations

This study has several limitations. It was cross-sectional and not designed to uncover changes in objective and subjective measures over time. The reported correlations are therefore not proof of causality.

As esophageal transit scintigraphy is not a routine clinical examination, there is no consensus on bolus composition, test conditions, or methods of analysis. Thus, the transit times are not directly comparable among studies.

Colonic transit time is presumably influenced by the consumption of laxatives and possibly by the intake of other medications that were not discontinued during the study. However, the use of laxatives among patients was significantly higher creating a bias towards the null hypothesis. No correlation was seen between the LEDD and colonic transit, which would have been expected if anti-Parkinsonian medications had pronounced effect on colonic transit. However, based on this study, it is not possible to clarify the effects of medications on gastrointestinal motility. Measures of dietary habits and activity level were not investigated, and the possible effect of these on colonic transit is therefore not accounted for.

In this study, the RBDSQ was used to evaluate symptoms of RBD, and although this questionnaire is validated, it is not equal to a full video-polysomnography, which is considered the gold standard for diagnosing RBD.

## 5. Conclusion

In conclusion, distal esophageal transit times and colonic transit times were both prolonged in early moderate PD. A highly significant correlation was found between RBD symptoms and colonic transit times. Notably, this correlation was stronger than between colonic transit time and symptoms of constipation. These findings support the tight link between iRBD and gastrointestinal dysfunction. Distal esophageal transit times did not correlate with colonic transit times or symptoms of dysphagia, but a link between subclinical esophageal dysfunction and weight loss might exist. Future longitudinal studies of the development of gastrointestinal dysfunction in iRBD and PD are needed to explore the development of objective esophageal and colonic dysfunction in different subtypes of PD.

## Figures and Tables

**Table 1:** Demographic, clinical, and scintigraphy data from patients with Parkinson disease (PD) and healthy controls (HC).

	PD patients (*n* = 30)	Healthy controls (*n* = 28)	*P* value
Demographical and clinical data			
Sex, male/female	20/10	18/10	0.99
Age, years	71 (66.6–74.6)	71.5 (66.9–75.8)	0.85
Height, cm	175.1 ± 9.5	173.5 ± 8.7	0.51
BMI, kg/m^2^	24.8 ± 3.6	25.7 ± 3.1	0.29
Time since diagnosis, years	6.5 ± 3.4	N/A	N/A
Motor symptoms duration, years	9.0 ± 4.0	N/A	N/A
Hoehn and Yahr stage (1/2/3)	4/23/3	N/A	N/A
MDS-UPDRS part III	23.7 ± 7.9	N/A	N/A
LEDD (mg of levodopa equivalents per day)	773.7 ± 272.1	N/A	N/A
Laxatives used (0/1/2/3/4 different types)	11/11/6/1/1	21/7/0/0/0	0.018
Orthostatic hypotension (yes/no)	14/13	0/27	<0.0001
ROM (number of markers)	31.3 ± 14.7	21.0 ± 9.1	0.002
Colonic transit time (days)	3.6 ± 1.5	2.6 ± 0.9	0.002
MoCA score	26.6 ± 1.8	27.5 ± 2.4	0.037
Sniffin' Sticks score	6.9 ± 2.84	12.6 ± 1.48	<0.0001
RBDSQ score	5.5 (4–8)	1 (0–2)	<0.0001
ROME III functional constipation	11 (5–23.5)	2 (0–4.8)	<0.0001
ROME III functional esophageal disorders	3.5 (0–10.3)	0 (0–3)	0.12
SCOPA-AUT	16.5 (13.8–22.3)	7.5 (5–10.8)	<0.0001
MDT-PD total points	10 (4.5–20.5)	0 (0–3)	<0.0001
MDT-PD weighted score	3.5 (2.3–5.1)	2.4 (2.4–3.0)	0.12

Algorithm-based analyses of esophageal scintigraphy			
Total transit (s)	5.47 (5.47–6.25)	5.77 (4.90–8.06)	0.47
Distal 6 centimeters (s)	2.23 (1.77–3.78)	1.88 (1.61–2.78)	0.05
Distal 4 centimeters (s)	2.09 (1.77–3.78)	1.56 (1.31–2.20)	<0.017
Distal 2 centimeters (s)	1.63 (1.02–2.49)	1.10 (0.77–1.53)	<0.040

Region of interest based analyses of esophageal scintigraphy			
Total transit, ROI 1–10 (s)	6.41 ± 1.45	6.01 ± 0.84	0.35
ROI 1–4	0.90 ± 0.31	0.78 ± 0.20	0.11
ROI 7–10	3.11 ± 0.73	2.89 ± 0.64	0.28
Upper part (total passage time, s)	1.65 ± 0.32	1.63 ± 0.31	0.86
Distal part (total passage time, s)	6.76 ± 2.91	5.42 ± 1.13	0.075

Data given as mean ± SD or median (interquartile range). N/A, not applicable; BMI, body mass index; MDS-UPDRS, Movement Disorder Society-Unified Parkinson's Disease Rating Scale; LEED, levodopa equivalent daily dose; MoCA, Montreal cognitive assessment; RBDSQ, REM sleep behavior disorder screening questionnaire; MDT-PD, Munich dysphagia test-Parkinson's disease.

## Data Availability

The data and the code developed for the esophageal transit algorithm used to support the findings of this study are available from the corresponding author upon request.

## Supplementary Materials

Supplementary Materials In the supplementary material, additional methodological details are provided concerning the esophageal scintigraphy. The material illustrates the applied definition of the gastroesophageal junction, the algorithm-based analyses, and the ROI-based methods for analysis of the esophageal scintigraphy.
