# Supplementary material for: Intestinal Transit in Early Moderate Parkinson's Disease Correlates with Probable RBD: Subclinical Esophageal Dysmotility Does Not Correlate
Source: Parkinsons Dis. 2022 Jul 15;2022:4108401. doi: 10.1155/2022/4108401 (PMC9307413; doi:10.1155/2022/4108401)
Supplement: Supplementary Materials — In the supplementary material, additional methodological details are provided concerning the esophageal scintigraphy. The material illustrates the applied definition of the gastroesophageal junction, the algorithm-based analyses, and the ROI-based methods for analysis of the esophageal scintigraphy. [file 4108401.f1.docx]

## Supplementary material

In this supplementary section additional methodological details are provided accompanied by image examples. The material will cover the applied definition of the gastroesophageal junction, the algorithm-based analyses and the ROI-based methods for analysis of the esophageal scintigraphy.

### The gastroesophageal junction

**
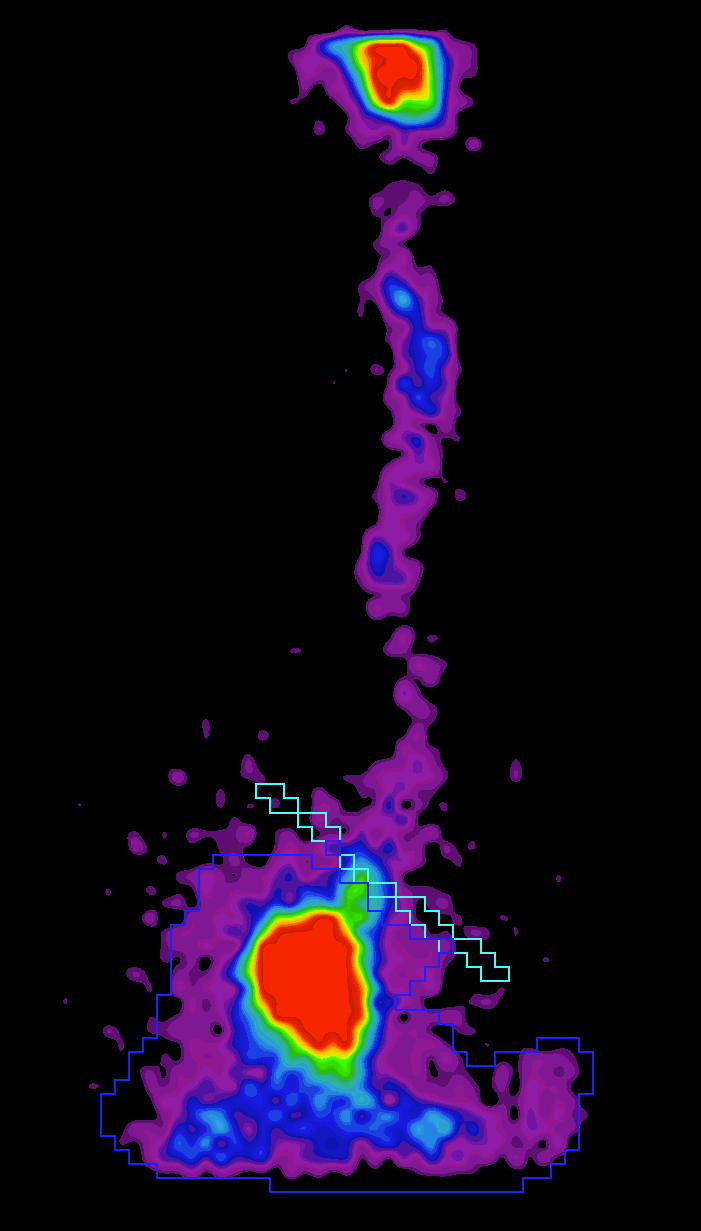
**Naturally, a clear definition of the gastroesophageal junction is needed to investigate the distal part of the esophagus without investigating the junction itself or the proximal part of the stomach. First, all subjects with suspected hiatal hernia were excluded based on CT imaging as described. Thereafter, 50 frames prior to swallowing of the last bolus for recording of the scintigraphy was summed to decide the area of radioactive accumulation corresponding to the stomach. Based on this a border was drawn and the coordinates of the crossing between this border and the distal esophagus was used as the standardized definition for the gastroesophageal junction (**Figure S1**).

**Figure S1:** Sum of an esophageal scintigraphy recording showing the area of radioactive accumulation marked with dark blue outline and the gastroesophageal border marked with light blue. Here, the summed imaged of the bolus swallow is also shown to visualize the esophagus.

### Algorithm-based approach

The software algorithm used for tracking the bolus transit though the distal esophagus on a per frame basis to enhance the ability to detect small differences in transit times. As such, the algorithm provided an estimate of the center of the bolus for each frame (8 frames per second) during the recording until the bolus reached the gastroesophageal junction (**Figure 1**).

### ROI-based approach

Two different methods based on regions of interest (ROIs) were used. The first used 10 ROIs of 20 x 30 mm spread evenly (**Figure S2A**) from the uppermost part of the esophagus to the gastroesophageal junction measuring the transit of the bolus by determining the time of peak activity in each bolus and afterwards subtract these measures to find the transit time between two ROIs. The second method used two larger ROIs (**Figure S2B**) placed corresponding to ROIs 1-3 (upper) and ROI 8-10 (distal) and the duration was measured from 20% of peak activity was first reached to 20% of peak activity again was reached.

**Figure S2A:** Regions-of-interest (ROIs) based analysis with smaller 10 ROIs. **Left**: A summed esophageal scintigraphy recording with placement of 10 ROIs on recording. **Right**: Time-activity curves showing the transit of the bolus though the esophagus. Each curve corresponds to the activity of one ROI. The time of maximal activity within each ROI was used for analysis.

**Figure S2B:** ROIs based analysis with two larger ROIs. **Left**: A summed esophageal scintigraphy recording with placement of two larger ROIs, a ROI covering the stomach and a line corresponding to the gastroesophageal junction. **Right:** Time-activity curves showing the transit of the bolus though the upper and lower ROI. The length of time from 20% of peak activity was reached until enough activity has passed to again reach 20% of peak activity was used.
